# Circular RNA circCSPP1 knockdown attenuates doxorubicin resistance and suppresses tumor progression of colorectal cancer via miR-944/FZD7 axis

**DOI:** 10.1186/s12935-021-01855-6

**Published:** 2021-03-04

**Authors:** Lanlan Xi, Quanlin Liu, Wei Zhang, Linshan Luo, Jingfeng Song, Ruitao Liu, Shue Wei, Yong Wang

**Affiliations:** 1 Department of Surgery of Traditional Chinese Medicine, Henan University of Traditional Chinese Medicine, Zhengzhou, China; 2 Department of Colorectal Surgery, Zhengzhou Anorectal Hospital, No. 51, Longhai East Road, Zhengzhou, 450004 China; 3 Department of Large Intestine, Zhengzhou Anorectal Hospital, Zhengzhou, China

**Keywords:** CircCSPP1, MiR-944, FZD7, DOX-resistant, Tumor progression, Colorectal cancer

## Abstract

**Background:**

Circular RNAs (circRNAs) have been reported to play vital roles in colorectal cancer (CRC). However, only a few circRNAs have been experimentally validated and functionally described. In this research, we aimed to reveal the functional mechanism of circCSPP1 in CRC.

**Methods:**

36 DOX sensitive and 36 resistant CRC cases participated in this study. The expression of circCSPP1, miR-944 and FZD7 were detected by quantitative real time polymerase chain reaction (qRT-PCR) and the protein levels of FZD7, MRP1, P-gp and LRP were detected by western blot. Cell proliferation, migration, invasion, and apoptosis were assessed by 3-(4, 5-dimethyl-2-thiazolyl)-2, 5-diphenyl-2-H-tetrazolium bromide (MTT) assay, transwell assay, or flow cytometry analysis, respectively. The interaction between miR-944 and circCSPP1 or frizzled-7 (FZD7) was predicted by Starbase 3.0 and verified by the dual luciferase reporter assay, RNA immunoprecipitation (RIP) assay and RNA pull down assay. Xenograft tumor assay was performed to examine the effect of circCSPP1 on tumor growth in vivo.

**Results:**

The expression of circCSPP1 and FZD7 was upregulated while miR-944 expression was downregulated in doxorubicin (DOX)-resistant CRC tissues and cells. CircCSPP1 knockdown significantly downregulated enhanced doxorubicin sensitivity, suppressed proliferation, migration, invasion, and induced apoptosis in DOX-resistant CRC cells. Interestingly, we found that circCSPP1 directly downregulated miR-944 expression and miR-944 decreased FZD7 level through targeting to 3′ untranslated region (UTR) of FZD7. Furthermore, circCSPP1 mediated DOX-resistant CRC cell progression and doxorubicin sensitivity by regulating miR-944/FZD7 axis. Besides, circCSPP1 downregulation dramatically repressed CRC tumor growth in vivo*.*

**Conclusion:**

Our data indicated that circCSPP1 knockdown inhibited DOX-resistant CRC cell growth and enhanced doxorubicin sensitivity by miR-944/FZD7 axis, providing a potential target for CRC therapy.

## Background

Colorectal cancer (CRC), a disease of modernity which killed nearly 700,000 people every year, has been the world’s fourth most deadly cancer [1, 2]. Despite many advances in the diagnosis and therapeutic improvements of this disease, the prognosis of CRC patients remains poor, owing to diagnosis is difficult in the earlier period and the occurrence of drug resistance [2, 3]. Doxorubicin (DOX), an anthracycline that inhibit nucleic acid synthesis [4], usually used to kill residual CRC cells after surgery and advanced colorectal cancer [5–7]. Despite the widely and long-term clinical application of DOX, current treatment effect is not very ideal on account of cell drug resistance [8]. DOX resistance is already a severe therapeutic impediment which directly affected the treatment effect and the lives of patients [8]. Therefore, a better understanding of the underlying mechanism of DOX resistance will has the enormous help for the treatment of CRC patients.

Circular RNAs (circRNAs), a class of endogenous noncoding RNAs with closed loop structures without 5′ caps and 3′ poly tails, play crucial roles in human cancer the initiation, progression and metastasis of tumors [9, 10]. Non-coding RNAs (ncRNAs) include microRNAs (miRNAs), long ncRNAs (lncRNAs), and circRNAs [11]. Mounting research has shown that miRNAs and lncRNAs are closely linked to multidrug resistance of cancers [12]. However, little is known about the role of circRNAs in drug resistance. Generally, previous studies have shown that circRNAs involved in the regulation of drug resistance in tumors by modulating various regulatory pathways and processes including the ceRNA regulatory network [13], EMT [14], cancer stem cells [15], apoptosis [16] and autophagy [17]. In colorectal cancer, Xiong et al. found that hsa_circ_0007031 and hsa_circ_0000504, promote 5-FU resistance by modulating the miR-885-3p/AKT3 and miR-485-5p/STAT3/AKT3 signaling pathways or by regulating the expression of BCL2 protein [13]. Hsa_circ_0005963 promotes glycolysis to induce chemoresistance by miR-122-PKM2 axis in colorectal cancer [18]. CircCSPP1 ((hsa_circ_0001806), derived from centrosome and spindle pole associated protein 1 (CSPP1), acts as an oncogene in luminal breast cancer [19] and human B-cell lymphoma [20]. Research has shown that circCSPP1 functions as a ceRNA to promote colorectal cancer cell EMT by upregulating COL1A1 [21]. Wang et al. found that circCSPP1 promotes tumorigenesis of colorectal cancer by sponging miR-1184 and releasing AJUBA and inactivating Hippo/YAP signaling [22]. Nonetheless, whether circCSPP1 participates in the process of chemoresistance in colorectal cancer still needs further research.

The current study is designed to improve our understanding of the precise mechanisms of DOX resistance in CRC, we detected the expression of circCSPP1, miR-944, and FZD7 in DOX-resistant CRC tissues and cells, and analyzed the function of circCSPP1 on DOX-resistant CRC cell progression, including proliferation, migration, invasion, and apoptosis. Furthermore, the effect of miR-944 and FZD7 on circCSPP1-mediated DOX-resistant cell progression was explored. Besides, we also investigated the effect of circCSPP1 on CRC tumor in vivo. Our findings will provide potentially novel target for the treatment of DOX resistant colorectal cancer.

## Materials and methods

### Tissues and cell culture

A total of 36 DOX sensitive or resistant tissues were obtained from chemo-sensitive or chemo-resistant patients from Zhengzhou Anorectal Hospital. All patients signed informed consents and did not receive any therapy.

Two cell lines (LoVo and HCT116) used in this research were provided by the American Type Culture Collection (ATCC, Manassas, VA, USA). LoVo and HCT116 cells were treated with increasing concentrations of DOX (0.01, 0.02, 0.04, 0.08, 0.16, 0.32, and 0.50 μg/mL) to establish the DOX-resistant LoVo and HCT116 cells (LoVo/DOX and HCT116/DOX). The DOX-resistant cells were grown in medium with 0.50 μg/mL DOX for maintenance of resistance. All the cells were cultured in RPMI-1640 medium (Gibco, Gaithersburg, MD, USA) containing 10% fetal bovine serum (FBS; HyClone, Logan, UT, USA) and incubated in a humidified incubator with 5% CO_2_ at 37 °C.

### Plasmid and transfection

CircCSPP1 overexpression vector, si-circCSPP1, miR-944 mimic, miR-944 inhibitor (in-miR-944) and their negative control (empty vectors) were provided by Genecopoeia (Guangzhou, China). To obtain the FZD7 overexpression vetor, FZD7 coding sequence (CDS) was amplified and inserted into the pcDNA 3.1 vector. Cell transfection in this research was performed using the Lipofectamine™ 2000 (Invitrogen, Carlsbad, CA, USA) and Opti-MEM (Gibco, Gaithersburg, MD, USA) according to the manufacturer's instructions.

### RNA extraction and quantitative real-time polymerase chain (qRT-PCR) assay

Total RNA was extracted from DOX-resistant CRC tissues or cells by using TRIzol (Invitrogen) regent according to the manufacturer's instructions. Quantification of miR-944 was performed by using TaqMan microRNA assay (Applied Biosystems; Thermo Fisher Scientific, Inc., Waltham, MA, USA). For analysis of CRNED and FZD7 expression, 1 µg of RNA was used to synthesize cDNA using Prime Script RT reagent Kit containing gDNA remover (Takara, Dalian, China). QRT-PCR was carried out in a volume of 10 µL using Go Taq qPCR Master Mix (Promega, Madison, WI, USA). For negative control groups, the cDNA in qRT-PCR was replaced by the double distilled H_2_O (ddH_2_O) under the same condition. U6 and glyceraldehyde 3-phosphate dehydrogenase (GAPDH) were chosen as reference genes in our experiment. The analysis of data was carried out using the 2^−ΔΔCT^ method [23]. For analysis of miR-944 expression, reverse transcription experiment was performed using specific stem-loop RT primer: 5′-CTCAACTGGTGTCGTGGAGTCGGCAATTCAGTTGAGCTCATCCG-3′.The primer sequences against circCSPP1, FZD7, U6, and GAPDH were listed as follows: circCSPP1 forward (F), 5′-CCATCCCATCAGTTCATCCT-3′ and reverse (R), 5′-CCCTGCAAAAGGACTACAGG-3′; FZD7 F, 5′-TTCTCGGACGATGGCTACC-3′ and R, 5′-GAACCAAGTGAGAGACAGAATGACC-3′; U6 F, 5′-TGCGGGTGCTCGCTTCGGCAGC-3′ and R, 5′-CCAGTGCAGGGTCCGAGGT-3′; GAPDH F, 5′-CATGAGAAGTATGACAACAGCCT-3′ and R, 5′-AGTCCTTCCACGATACCAAAGT-3’.

### Cell proliferation assay

2 × 10^3^ transfected LoVo/DOX and HCT116/ DOX cells were seeded into each well of 96-well plates and incubated for 24 h. 5 mg/mL 3-(4, 5-dimethyl-2-thiazolyl)-2, 5-diphenyl-2-H-tetrazolium bromide (MTT; Shguduo Biomart Inc., Shanghai, China) was added and incubated at 37  C for 4 h. Subsequently, 200 µL dimethylsulfoxide was used for dissolution of formazan product. Finally, the absorbance was measured by an ELISA plate reader (Bio-Rad Laboratories, Inc., Hercules, CA, USA) at 490 nm.

### Cell migration and invasion assay

Cell migration or invasion was detected by using a transwell chamber (Millipore, Billerica, MA, USA). For the transwell migration assay, the cells were seeded in the uncoated upper chamber with 100 µL medium without serum, while 500 µL corresponding medium containing 10% FBS was added into the lower chamber. For the invasion assay, matrigel (BD Biosciences, San Jose, CA, USA) was coated into the upper chamber and the bottom chambers were incubated overnight in 500 μL DMEM culture medium with 10% FBS. After cultured for 24 h, the migratory or invasive cells were stained by 1% crystal violet and counted using a microscope.

### Cell apoptosis assay

The cell apoptosis rate was detected by a propidium iodide (PI)/Annexin V-Fluorescein (FITC) kit (Solarbio, Beijing, China) and analyzed by flow cytometry. In brief, the transfected cells were harvested and washed by 1 × Binding Buffer for 2 times after transfection. Then, cells were stained with 10 µL Annexin V-FITC and 5 µL PI at the room temperature under the dark condition. Finally, flow cytometry (FACScan; Becton Dickinson, Franklin, NJ) was used to analyze cell apoptosis rate.

### Western blot assay

Total proteins were collected from DOX-resistant CRC cells using the RIPA buffer (Cell Signaling Technology, Boston, MA, USA) and 30 µg protein was subjected to dodecyl sulfate, sodium salt-Polyacrylamide gel electrophoresis (SDS-PAGE, 10%). Next, proteins were transferred to PVDF members (Millipore) and then blocked in TBST (Solarbio) containing 5% non-fat skim milk. Subsequently, the membranes were incubated with the primary antibodies against FZD7, multidrug resistance protein 1 (MRP1), P-glycoprotein (P-gp), lung resistance protein (LRP) and β-actin (1:1000; Abcam, Cambridge, MA, USA) overnight at 4 ℃ and then incubated with corresponding secondary antibodies (1:500; Abcam) at room temperature for 1 h. Final, protein signals were determined using the ECL plus Kit (Beyotime, Haimen, China).

### Dual luciferase reporter assay

The full or mutant fragments containing the predicted miR-944 binding sites in circCSPP1 or FZD7 3′UTR were cloned into the downstream of firefly luciferase gene in pGL3 vector (Promega, Fitchburg, WI, USA). Then, the DOX-resistant LoVo and HCT116 cells were co-transfected miR-944 or miR-NC with circCSPP1 WT or circCSPP1 WT, or FZD7 3′UTR-WT or FZD7 3′UTR-MUT. After incubation for 24 h, luciferase activity was measured by the Dual-Luciferase Reporter assay system (Promega).

### RNA immunoprecipitation (RIP) assay

RIP assay in this study was performed using Magna RNA immunoprecipitation kit (Millipore) according to the user’s manual. Briefly, after transfected with miR-NC or miR-944, LoVo/DOX or HCT116/DOX cells were cultured for 48 h and lysed by RIP buffer containing magnetic beads conjugated with Ago2 or IgG antibody. Then, immunoprecipitated RNAs were obtained using TRIzol reagent and enrichment of circCSPP1 was analyzed by qRT-PCR assay.

### RNA Pull down assay

The biotinylated circCSPP1 WT, circCSPP1 MUT, or NC was transfected into LoVo/DOX and HCT116/DOX cells. After cultured for 48 h, the cells were harvested, lysed by RIP buffer, and then incubated by streptavidin magnetic beads (M-280; Invitrogen) based on the manufacture’s manual. After incubated at cold room (4 ℃) for 2 h, the beads were washed by cold buffer for 3 times. Final, the RNAs was eluted for further experiments.

### Mouse xenografts

The cells transfected with sh-circCSPP1 or sh-NC were injected into 6-week-old nude mice subcutaneously. Tumor volume (length × width × width/2) was measured every 7 days. After injection for 5 weeks, the mice were executed and the tumor weight was calculated and tumors were stored at − 80 ℃ for further research.

## Statistical analysis

All data were based on at least three independent biological reduplications and demonstrated as mean ± standard deviation (SD). Student’s *t*-test was carried out to measure the difference between two groups and one-way ANOVA was used to measure the difference between more than two groups. *P* less than 0.05 was considered as statistical difference.

## Results

### CircCSPP1 expression was increased in DOX-resistant CRC tissues and cells

**Fig. 1:**
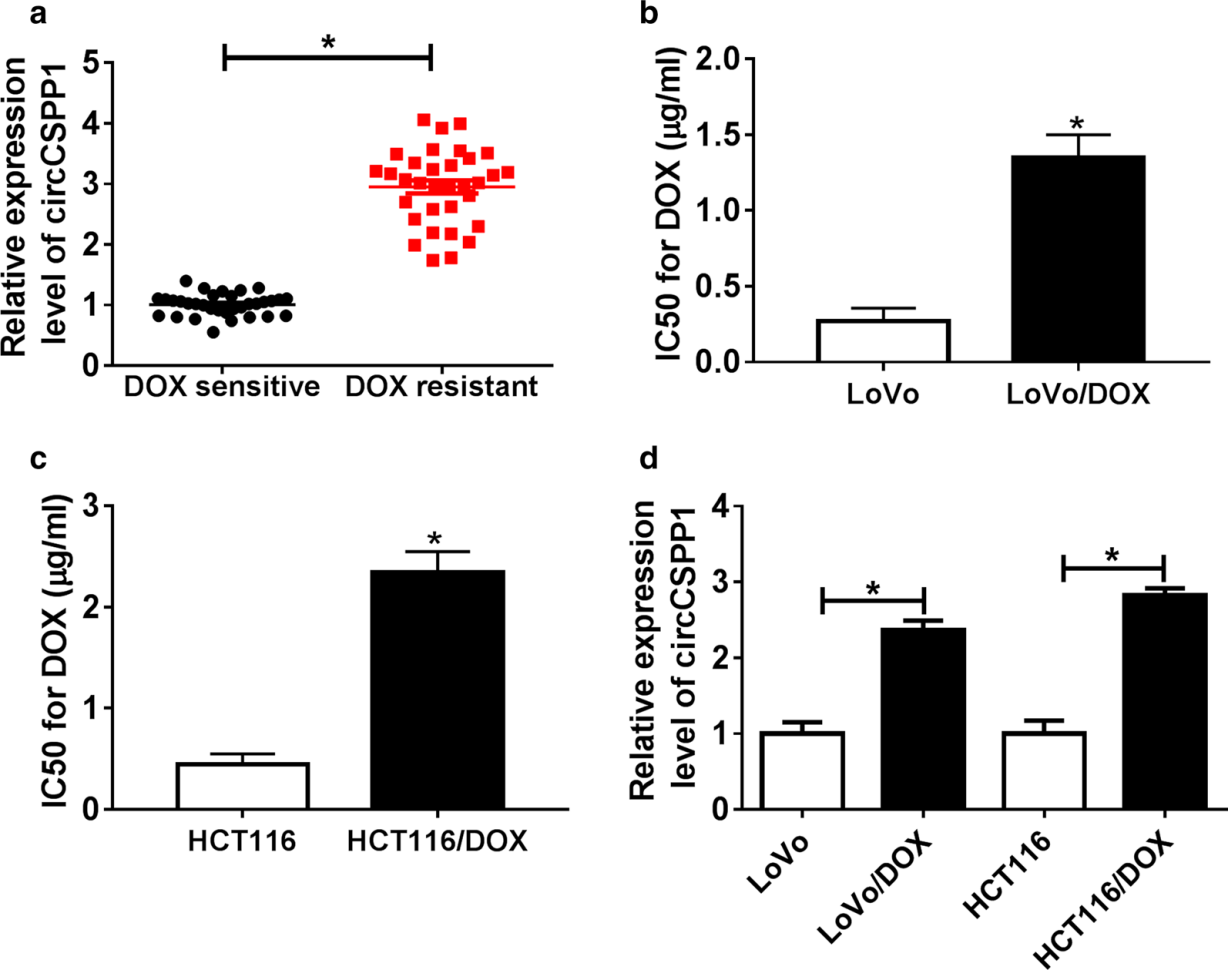
The expression level of circCSPP1 in DOX-resistant tissues and cells. **a** The expression level of circCSPP1 was detected by qRT-PCR in DOX-resistant or DOX-sensitive CRC tissues. **b**, **c** The DOX-resistant CRC LoVo/DOX (**b**) and HCT116/DOX (**c**) cells were analyzed by MTT assay. IC50 of 24 h was shown. **d** The expression of circCSPP1 was examined by qRT-PCR in LoVo/DOX and HCT116/DOX cells as well as in LoVo and HCT116 cells. *P < 0.05

To explore the roles of circCSPP1 in DOX-resistant CRC, we first detected the expression of circCSPP1 in DOX-resistant CRC tissues and cells. As shown in Fig. 1a, circCSPP1 expression was significantly upregulated in DOX-resistant CRC tissues. To detect the expression of circCSPP1 in DOX-resistant CRC cells, DOX-resistant CRC LoVo and HCT116 cell model (LoVo/DOX and HCT116/DOX) were constructed. As shown Fig. 1b, c, the IC50 of LoVo/DOX and HCT116/DOX cells were significantly improved compared to LoVo and HCT116 cells, which indicated the successfully established of DOX-resistant CRC LoVo and HCT116 cell model. Then, we further analyzed circCSPP1 expression in LoVo/DOX and HCT116/DOX cells, and found that the expression of circCSPP1 in DOX-resistant CRC cells was significantly higher than that in normal CRC cells (Fig. 1d). These data indicated that the abnormal expression of circCSPP1 is closely related to DOX resistance of CRC cells.

### CircCSPP1 knockdown enhanced DOX sensitivity, suppressed cell proliferation, migration, and invasion, as well as promoted apoptosis in DOX-resistant CRC cells

**Fig. 2:**
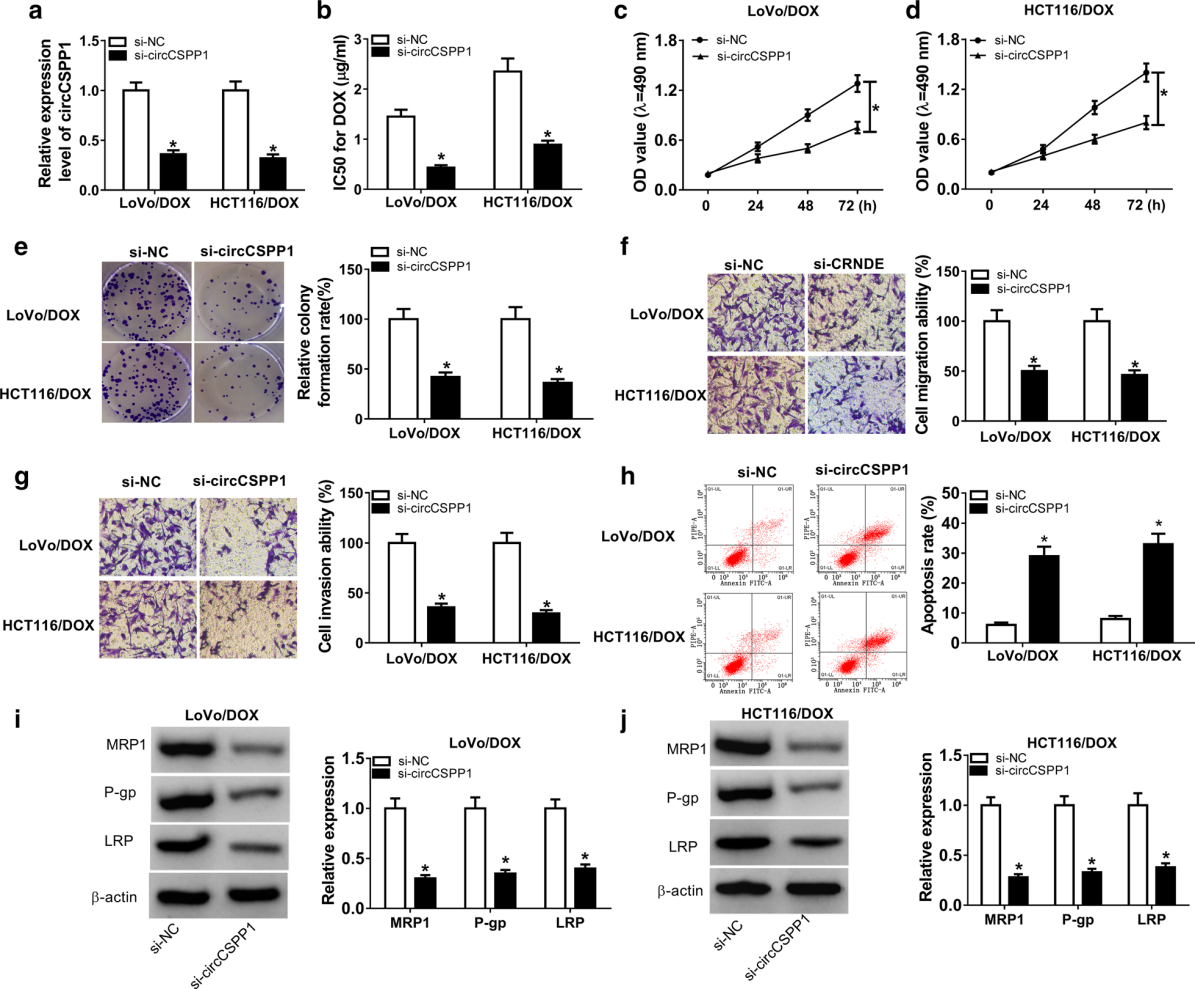
CircCSPP1 knockdown enhanced DOX sensitivity, inhibited cell proliferation, migration, invasion, and promoted apoptosis in DOX-resistant CRC cells. **a–j** LoVo/DOX and HCT116/DOX cells were transfected with si-NC or si-circCSPP1, respectively. **a** CircCSPP1 expression was determined by qRT-PCR in LoVo/DOX and HCT116/DOX cells after transfection. **b** The transfected LoVo/DOX and HCT116/DOX cells were analyzed by MTT assay. IC50 of 24 h was shown. **c**, **d** Cell proliferation was measured by MTT assay in LoVo/DOX (**c**) and HCT116/DOX (**d**) cells after transfection for 0 h, 24 h, 48 h, and 72 h respectively. **e** Cell proliferation was detected by colony formation assay. **f**, **g** Transwell assay was performed to assess cell migration (**e**) and invasion (**f**). **h** Cell apoptosis rate was determined by flow cytometry analysis. **i**, **j** Western blot assay was carried out to detect the protein levels of MRP1, P-gp and LRP. *P < 0.05

In order to further investigate the function of circCSPP1 in DOX-resistant CRC cells, circCSPP1 knockdown was performed by transfection of si-circCSPP1. QRT-PCR analysis showed that si-circCSPP1 remarkably decreased the expression of circCSPP1 in LoVo/DOX and HCT116/DOX cells (Fig. 2a). Subsequently, the effects of circCSPP1 knockdown on DOX-resistant CRC cells was analyzed. As demonstrated in Fig. 2b, circCSPP1 knockdown significantly decreased IC50 of DOX-resistant CRC cells. MTT assay suggested that cell proliferation ability in circCSPP1-depleted LoVo/DOX and HCT116/DOX cells (Fig. 2c, d) was lower than that in control cells. Besides, the decreased colony formation rate (Fig. 2e) in DOX-resistant CRC cells with circCSPP1 knockdown also confirmed that cell proliferation ability of DOX-resistant CRC cells was inhibited by si-circCSPP1. Meanwhile, we also explored cell migration and invasion abilities after circCSPP1 depletion, and the results showed that circCSPP1 downregulation dramatically suppressed the migration (Fig. 2f) and invasion (Fig. 2g) of DOX-resistant CRC cells. Besides, cell apoptosis rate was analyzed by using flow cytometry. Increased cell apoptosis was observed in circCSPP1-depleted DOX-resistant CRC cells (Fig. 2h). Besides, the protein levels of drug resistance-related protein MRP1, P-gp and LRP were detected by western blot assay. As shown in Fig. 2i, j, the levels of MRP1, P-gp and LRP protein were strongly downregulated by circCSPP1 knockdown in LoVo/DOX and HCT116/DOX cells. Therefore, circCSPP1 depletion suppressed DOX resistance, inhibited proliferation, migration, and invasion, and induced apoptosis in DOX-resistant CRC cells.

### CircCSPP1 directly targets to miR-944 and negatively regulated miR-944

**Fig. 3:**
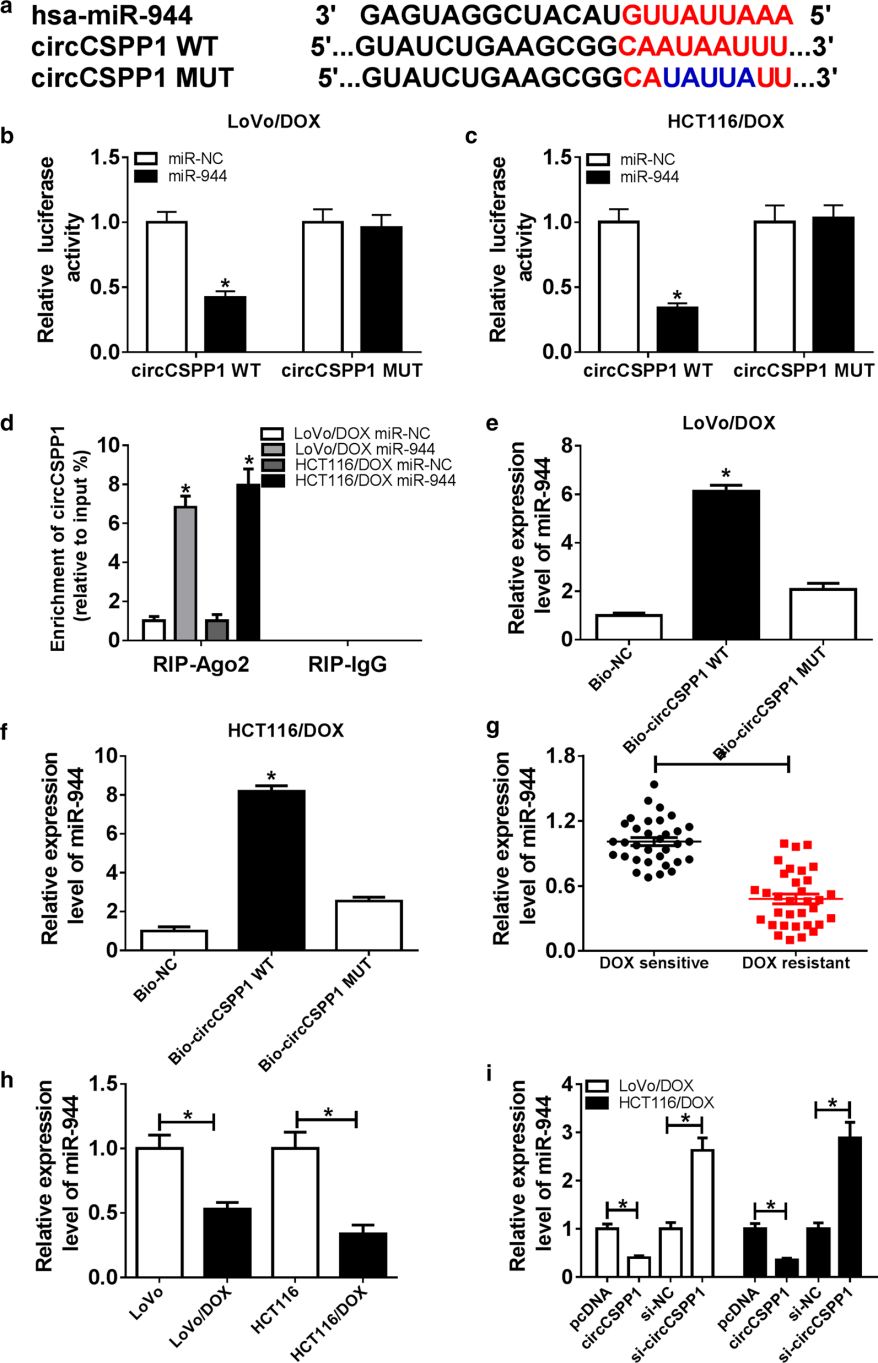
CircCSPP1 targets to miR-944. **a** Potential targets of circCSPP1 were predicted by DIANA tool. The red trace is the predicted binding sites and the blue mark represents the circCSPP1 mutation sites. **b, c** Relative luciferase activity was determined in both LoVo/DOX (**b**) and HCT116/DOX cells (**c**) co-transfected with miR-944 or miR-NC and circCSPP1 WT or circCSPP1 MUT. **d–f** RIP (**d**) and RNA pull down (**e**, **f**) assays were conducted to confirm the interaction between circCSPP1 and miR-944. **g, h** miR-944 expression was detected by qRT-PCR assay in DOX-resistant and DOX-sensitive CRC tissues (**g**) or DOX-resistant and normal CRC cells (**h**). **i** MiR-944 expression was measured by qRT-PCR in cells transfected with pcDNA, circCSPP1, si-NC, and si-circCSPP1, respectively. *P < 0.05

Bioinformatics analysis tool DIANA revealed that circCSPP1 might target to miR-944 (Fig. 3a). Dual-luciferase reporter assay, RIP assay and RNA pull down assay were performed to verify the forecast results. As shown in Fig. 3b, c, relative luciferase activity was decreased when cells were co-transfected with circCSPP1 WT and miR-944, while it has little change in cells transfected with circCSPP1 MUT and miR-944. Endogenous circCSPP1 was specifically enriched by anti-AGO2 antibody (Fig. 3d). And RNA pull down assay showed that wild-type circCSPP1 captured more miR-944 compared with the mutant (Fig. 3e, f) in DOX-resistant CRC cells, indicating that circCSPP1 directly targets to miR-944. Besides, the expression of miR-944 was detected in DOX-resistant CRC tissues and cells. The data demonstrated that miR-944 expression was decreased in DOX-resistant CRC tissues (Fig. 3g) and cells (Fig. 3h). In addition, we analyzed the effect of circCSPP1 on miR-944 expression, and found that transfection of circCSPP1 downregulated the expression level of miR-944 while the expression level of miR-944 was upregulated in LoVo/DOX and HCT116/DOX cells transfected with si-circCSPP1 (Fig. 3i). These data suggested that circCSPP1 negatively regulated miR-944 expression.

### CircCSPP1 modulated cell progression by regulating miR-944 expression

**Fig. 4:**
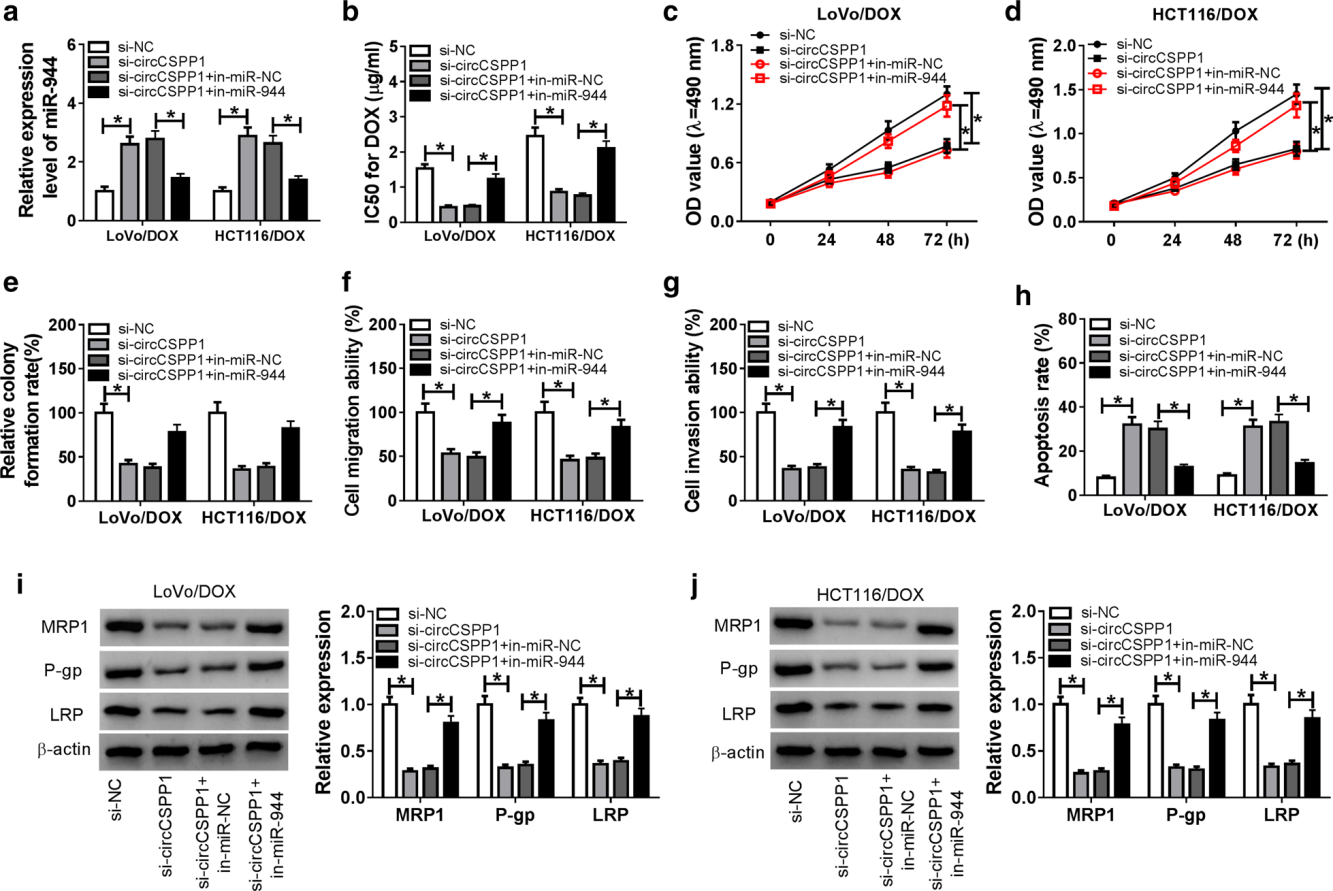
CircCSPP1 contributes to cell progression and DOX-resistant by sponging miR-944. **a–j** LoVo/DOX and HCT116/DOX cells were transfected with si-NC, si-circCSPP1, si-circCSPP1 + in-miR-NC, or si-circCSPP1 + in-miR-944, respectively. **a** MiR-944 expression was detected by qRT-PCR in transfected LoVo/DOX and HCT116/DOX cells. **b** The transfected LoVo/DOX and HCT116/DOX cells were analyzed by MTT assay. IC50 of 24 h was shown. **c–e** Cell proliferation was assessed by MTT assay **c, d** and colony formation assay (**e**). **f, g** Transwell assay was performed to analyze cell migration (**f**) and invasion (**g**). **h** Flow cytometry analysis was used to examine cell apoptosis. **i, j** The protein levels of MRP1, P-gp and LRP were measured by western blot assay. *P < 0.05

As circCSPP1 downregulated miR-944 expression in DOX-resistant CRC cells, we speculated that circCSPP1 exerted its function by regulating miR-944. To validate this assumption, LoVo/DOX and HCT116/DOX cells were transfected with si-NC, si-circCSPP1, si-circCSPP1 + in-miR-NC, or si-circCSPP1 + in-miR-944 respectively. QRT-PCR analysis verified that circCSPP1 knockdown significantly increased miR-944 expression, while in-miR-944 reversed the increase of miR-944 induced by circCSPP1 depletion (Fig. 4a). Besides, miR-944 depletion elevated the value of IC50 that was downregulated by circCSPP1 knockdown in LoVo/DOX and HCT116/DOX cells (Fig. 4b). MTT assay (Fig. 4c, d) and colony formation assay (Fig. 4e) showed that transfection of si-circCSPP1 significantly inhibited cell proliferation, while this inhibition effect was reversed by in-miR-944 in LoVo/DOX and HCT116/DOX cells. Then the effects of miR-944 on circCSPP1-regulated DOX-resistant CRC cell migration, invasion, and apoptosis were analyzed through transwell assay and flow cytometry analysis, respectively. The results suggested that the downregulation of miR-944 hindered effects of circCSPP1 knockdown on cell migration (Fig. 4f), invasion (Fig. 4g), and apoptosis (Fig. 4h). Besides, circCSPP1-depleted inhibited the protein level of MRP1, P-gp and LRP, while increased protein levels were observed in cells co-transfected with si-circCSPP1 and in-miR-944 (Fig. 4i, j). Thus, circCSPP1 mediated cell progression and DOX-sensitivity by modulation of miR-944 expression in DOX-resistant CRC cells.

### MiR-944 downregulated FZD7 expression via targeting 3′ UTR of FZD7

**Fig. 5:**
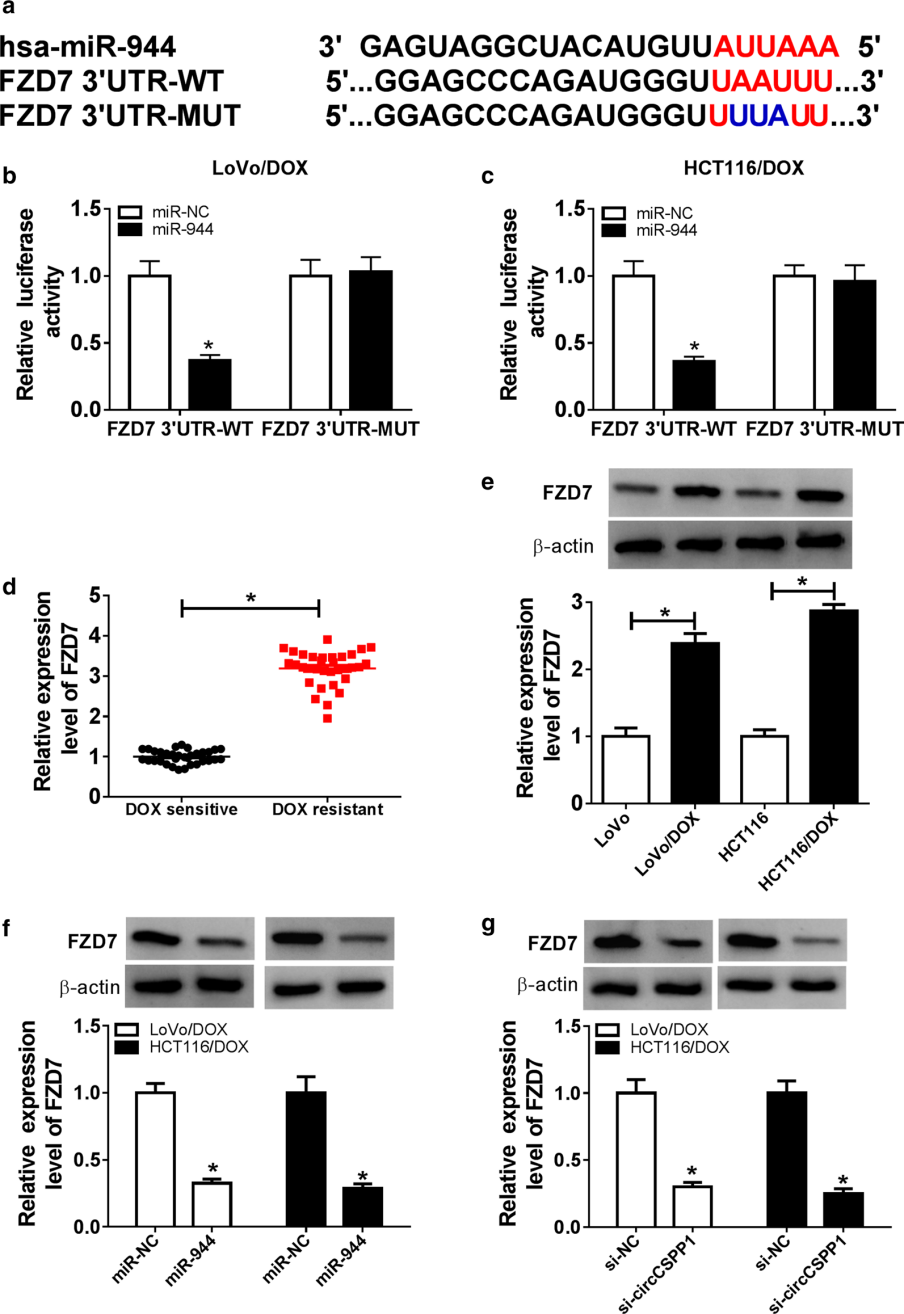
MiR-944 directly target to FZD7. **a** DIANA tool was used to predict potential target genes of miR-944. The red trace is the predicted binding sites and the blue mark represents the mutant sites in FZD7 3′-UTR. **b, c** Relative luciferase activity was determined in both LoVo/DOX and HCT116/DOX cells co-transfected with miR-944 or miR-NC and FZD7 3′UTR WT or FZD7 3′UTR MUT. **d, e** The mRNA and protein level of FZD7 was detected by qRT-PCR assay in DOX-resistant and DOX-sensitive CRC tissues (**d**) or DOX-resistant and normal CRC cells (**e**). **f, g** Western blot assay was performed to measure the protein level of FZD7 in cells transfected with miR-944 (**f**) or si-circCSPP1 (**g**). *P < 0.05

FZD7 was predicted as a potential target of miR-944 through bioinformatics analysis tool DIANA (Fig. 5a). Dual luciferase reporter assay displayed that relative luciferase activity of FZD7 3′ UTR-WT, but not FZD7 3′ UTR-MUT, was decreased by miR-944 in LoVo/DOX and HCT116/DOX cells (Fig. 5b, c). Thus, miR-944 directly bound to 3′ UTR of FZD7. Next, FZD7 expression in DOX-resistant CRC tissues and cells was detected by qRT-PCR assay. As shown in Fig. 5d, e, FZD7 level was significantly increased in DOX-resistant CRC tissues and cells. Furthermore, miR-944 significantly downregulated the expression level of FZD7 in cells transfected with miR-NC or miR-944 (Fig. 5f). Besides, decreased FZD7 level was also observed in cells transfected with si-circCSPP1 (Fig. 5g). Taken together, miR-944 directly downregulated FZD7 expression.

### MiR-944 mediated DOX-resistant CRC cell progression and DOX resistant by regulation of FZD7 expression

**Fig. 6:**
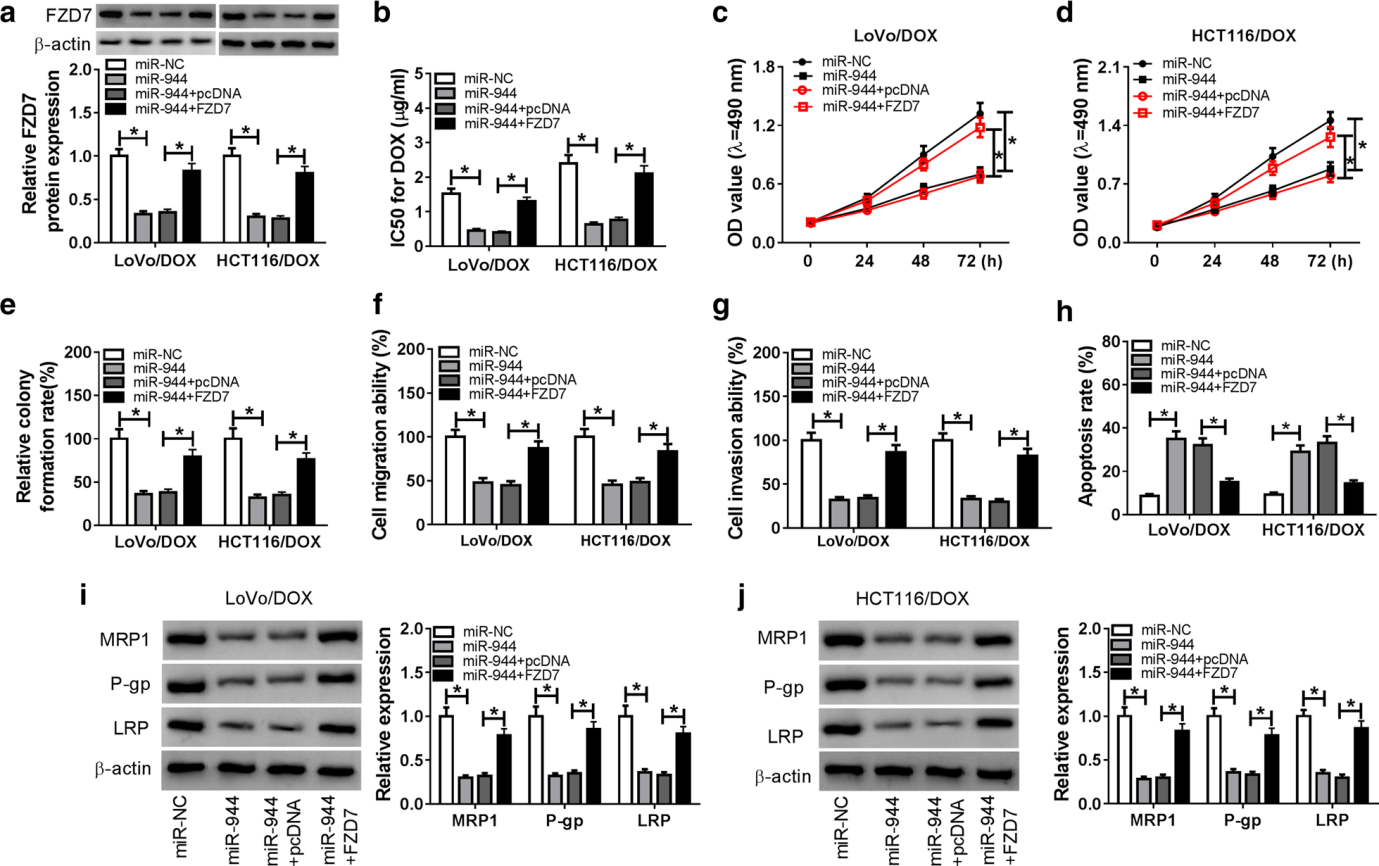
FZD7 overexpression partly reversed the effects of miR-944 on DOX-resistant CRC cells. **a–j** LoVo/DOX and HCT116/DOX cells were transfected with miR-NC, miR-944, miR-944 + pcDNA, or miR-944 + FZD7, respectively. **a** The mRNA and protein levels of FZD7 were detected in transfected LoVo/DOX and HCT116/DOX cells. **b** The transfected LoVo/DOX and HCT116/DOX cells were analyzed by MTT assay. IC50 of 24 h was shown. (**c–e**) Cell proliferation was determined by MTT assay after transfection for 0 h, 24 h, 48 h, and 72 h (**c, d**) or colony formation assay (**e**). **f, g** Cell migration (**f**) and invasion (**g**) were assessed using a transwell assay. **h** Cell apoptosis rate was analyzed with flow cytometry analysis. **i,** **j** Western blot assay was performed to detect the protein levels of MRP1, P-gp and LRP. *P < 0.05

Based on the above results, we speculate that miR-944 regulated FZD7 expression to modulate DOX-resistant CRC cell progression. Then, miR-NC, miR-944, miR-944 + pcDNA, or miR-944 + FZD7 was transfected into LoVo/DOX and HCT116/DOX cells respectively to confirm this hypothesis. QRT-PCR and western blot assays were performed to detect the level of FZD7. As indicated in Fig. 6a, the protein level and mRNA level of FZD7 was downregulated by miR-944 and partly rescued by transfection of FZD7. Besides, the IC50 value in cells transfected with miR-944 was significantly decreased, and it was increased in cells co-transfected with miR-944 and FZD7 (Fig. 6b). Besides, miR-944 inhibited cell proliferation (Fig. 6c–e), migration (Fig. 6f), invasion (Fig. 6g), and induced apoptosis (Fig. 6h), while transfection of FZD7 reversed the effects of miR-944 on DOX-resistant cells (Fig. 6f–h). Besides, miR-944 modulated FZD7 expression to regulate drug resistance-related protein expression was also observed (Fig. 6i, j). These data showed that miR-944 modulated DOX-resistant cell progression and DOX-resistant via regulating FZD7 expression in DOX-resistant CRC cells.

### CircCSPP1 depletion repressed tumor growth in vivo

**Fig. 7:**
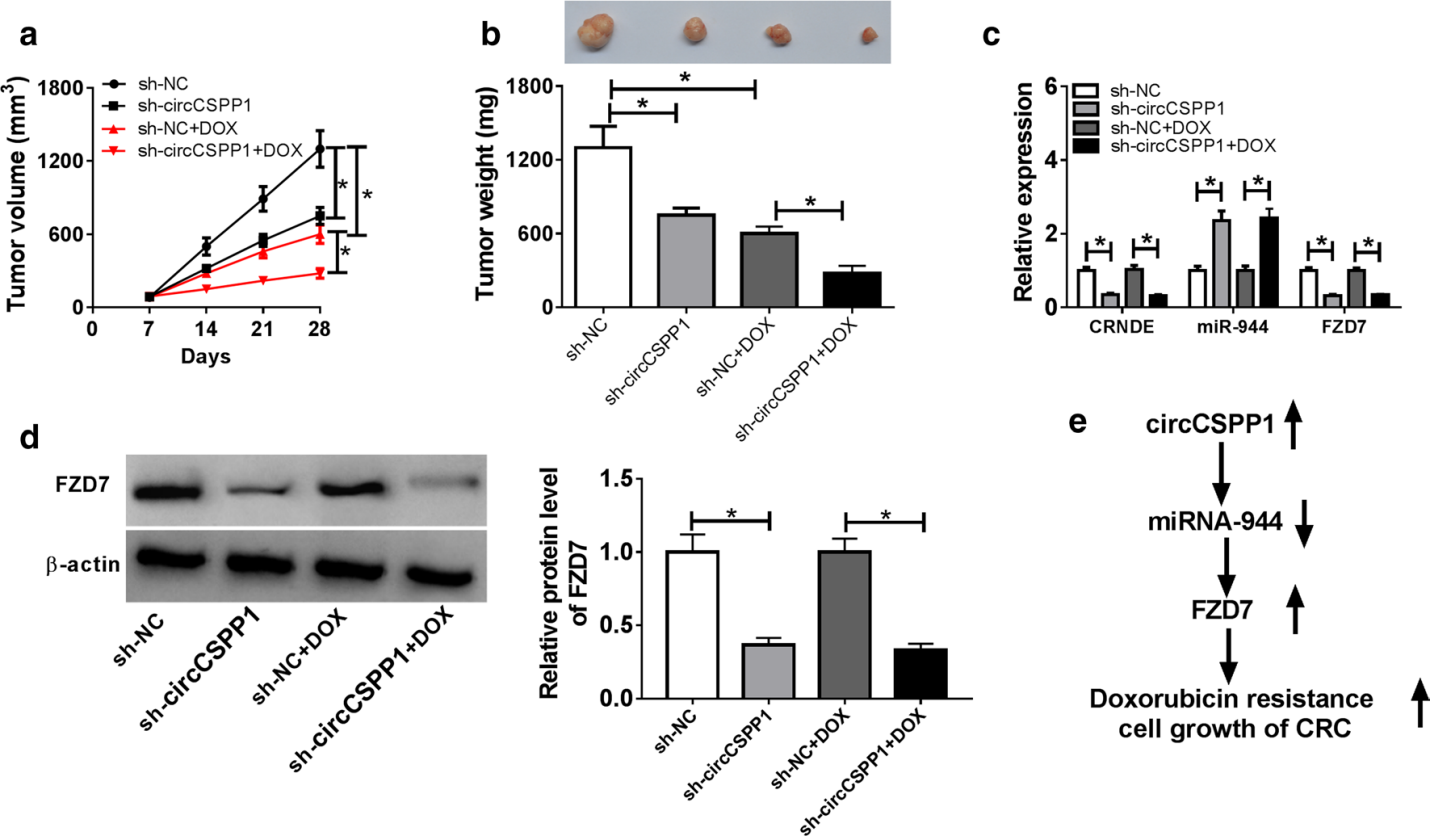
CircCSPP1 knockdown suppresses the tumor growth of CRC in vivo. **a–d** The nude mice were injected with CRC cells with stable expression of sh-NC or sh-circCSPP1 and treated with DOX. **a** Tumor volume was measured every 7 days to 28 days. **b** After injection for 28 days, the mice were executed and tumors were weighted. **c** The expression of circCSPP1, miR-944, and FZD7 in tumors were detected by qRT-PCR assay. **d** The protein level of FZD7 was detected by western blot assay. **d** Diagram of circCSPP1 regulated DOX resistance and tumor progression. *P < 0.05

To investigate the function of circCSPP1 in CRC tumorigenesis in vivo, nude mice were subcutaneously injected with LoVo/DOX cells stable expressed sh-NC or sh-circCSPP1, and then treated with DOX. Next, the tumor volume was measured every 7 days to 28 days. The mice were executed and tumors were weighted after injection for 28 days. The data demonstrated that tumor volume (Fig. 7a) and weight (Fig. 7b) were significantly inhibited by circCSPP1 depletion, and strengthened the treatment effect of DOX. Besides, qRT-PCR assay was performed to examine the expression levels of circCSPP1, miR-944, and FZD7. As expected, circCSPP1 and FZD7 levels were downregulated and miR-944 level was upregulated in circCSPP1-depleted cells (Fig. 7c, d). These data suggested that the depletion of circCSPP1 suppressed CRC tumor growth in vivo.

## Discussion

Herein, circCSPP1 was found to be overexpressed in DOX-resistant CRC tissues and cells. Knockdown of circCSPP1 enhanced DOX sensitivity, suppressed cell proliferation, migration, and invasion, as well as promoted apoptosis in DOX-resistant CRC cells and inhibited cell growth in vivo. MiR-944 is a target of circCSPP1 and directly regulated the expression of FZD7. Our data confirmed that circCSPP1 modulated DOX-resistant CRC cell progression and DOX sensitivity by regulating miR-944/FZD7 axis. These findings provided a potential target for the treatment of CRC.

Numerous studies has proved that circRNAs play a vital role in various human tumor occurrence and development including colorectal cancer [10, 24]. However, biological functions of circRNAs in colorectal cancer, especially in drug resistance, is just beginning. Therefore, in the present study, our research focused on the roles of circCSPP1 in DOX-resistant colorectal cancer. CircCSPP1, arise from centrosome and spindle pole associated protein 1, was upregulated in colorectal cancer [21, 22]. Research has shown that circCSPP1 acts as a promising therapeutic target by regulating the EMT process in colorectal carcinoma via activation of the circCSPP1/miR-193a-5p/COL1A1 axis [21]. Besides, CircCSPP1 could promote tumorigenesis of colorectal cancer by sponging hsa-miR-1184 and releasing AJUBA and inactivating Hippo/YAP signaling [22]. Consistent with the results described in previous research, circCSPP1 was upregulated in DOX-resistant CRC tissues and cells. The expression of circCSPP1 is closely related to DOX sensitivity, cell proliferation, migration, invasion and apoptosis in DOX-resistant CRC cells, implying the pivotal role of circCSPP1 in DOX-resistant CRC.

It is widely accepted that circRNA, lncRNA, and mRNA share the same miRNA response elements (MREs), binding to miRNAs and enhancing the expression of each other [25]. MiRNAs, defined as a small non-coding RNAs, are a class of important regulator that play pivotal roles in downstream gene expression by binding to 3′ untranslated region (UTR) of genes or promoting mRNA degradation [26, 27]. Otherwise, accumulating evidence suggested that miRNAs were involved in cell growth and drug resistance, and considered as a diagnostic biomarkers [28–30]. In this study, we found that miR-944 was a target gene of circCSPP1 by prediction using bioinformatics analysis and verification using the dual luciferase reporter, RIP, and RNA pull down assays. Previous research has shown that miR-944 upregulation inhibited cell progression, including proliferation and migration, through mediating GATA binding protein 6 (GATA6) level in CRC [31]. Wen et al. also verified that miR-944 suppressed CRC cell growth via regulation of p53 signaling [32]. These data revealed that miR-944 played a crucial role in CRC development. Moreover, we also confirmed that circCSPP1 negatively regulated miR-944 expression. Combining to circCSPP1 functions and the effect of circCSPP1 on miR-944 expression, we speculated that circCSPP1 modulated miR-944 expression to mediated CRC cell progression. And our data demonstrated that miR-944 knockdown reversed the effects of circCSPP1 depletion on DOX-resistant CRC cell proliferation, migration, invasion, and apoptosis.

Present studies revealed that miRNAs acted as mRNA sponges to repress the expression of these mRNAs [33]. Online tool DIANA was supplied to predict potential targets of miR-944. We found that miR-944 binding to the 3′-UTR of FZD7 and verified this interaction by the dual luciferase reporter assay. FZD7, a member of the FZD family, regulates tumor metastasis through modulating canonical and non-canonical Wnt pathway [34, 35]. FZD7 played important roles in various human cancers containing CRC [36–39]. Previous results demonstrated that FZD7 knockdown suppressed cell viability and invasion in CRC [38]. Elizabeth et al*.* proved that FZD7 expression, mediated by β-catenin/TCF4, was negatively modulated at the invasive front of CRC [40]. These results indicated that FZD7 positively regulated CRC development. In accordance with these data, the results in this research suggested that FZD7 overexpression induced DOX-resistant CRC cell development suppressed by miR-944. Taken together, circCSPP1 regulated DOX-resistant CRC cell progression via modulation of miR-944/FZD7 axis.

## Conclusion

In summary, circCSPP1 knockdown enhanced DOX sensitivity, attenuated cell proliferation, migration, and invasion, as well as induced apoptosis via regulating miR-944/FZD7 axis in DOX-resistant CRC cells, providing novel value for the therapy of CRC.

## Data Availability

The data sets used and/or analyzed during the current study are available from the corresponding author on reasonable request.
